# Skin collagen fluorophore LW-1 versus skin fluorescence as markers for the long-term progression of subclinical macrovascular disease in type 1 diabetes

**DOI:** 10.1186/s12933-016-0343-3

**Published:** 2016-02-11

**Authors:** David R. Sell, Wanjie Sun, Xiaoyu Gao, Christopher Strauch, John M. Lachin, Patricia A. Cleary, Saul Genuth, The DCCT/EDIC Research Group, Vincent M. Monnier

**Affiliations:** Department of Pathology, Case Western Reserve University, Wolstein Research Bldg. 5-301, 2103 Cornell Road, Cleveland, OH 44106 USA; Biostatistics Center, George Washington University, Rockville, MD 20852 USA; Department of Medicine, Case Western Reserve University, Cleveland, OH 44106 USA; Department of Biochemistry, Case Western Reserve University, Cleveland, OH 44106 USA

**Keywords:** Glycation, Skin autofluorescence, HbA1c, Retinopathy, Nephropathy, Neuropathy

## Abstract

**Background:**

Skin collagen Long Wavelength Fluorescence (LWF) is widely used as a surrogate marker for accumulation of advanced glycation end-products. Here we determined the relationship of LWF with glycemia, skin fluorescence, and the progression of complications during EDIC in 216 participants from the DCCT.

**Methods:**

LW-1 and collagen-linked fluorescence (CLF) were measured by either High Performance Liquid Chromatography (HPLC) with fluorescence detection (LW-1) or total fluorescence of collagenase digests (CLF) in insoluble skin collagen extracted from skin biopsies obtained at the end of the DCCT (1993). Skin intrinsic fluorescence (SIF) was noninvasively measured on volar forearm skin at EDIC year 16 by the SCOUT DS instrument.

**Results:**

LW-1 levels significantly increased with age and diabetes duration (P < 0.0001) and significantly decreased by intensive *vs.* conventional glycemic therapy in both the primary (P < 0.0001) and secondary (P < 0.037) DCCT cohorts. Levels were associated with 13–16 year progression risk of retinopathy (>3 sustained microaneurysms, P = 0.0004) and albumin excretion rate (P = 0.0038), the latter despite adjustment for HbA_1c_. Comparative analysis for all three fluorescent measures for future risk of subclinical *macrovascular disease* revealed the following significant (P < 0.05) associations after adjusting for age, diabetes duration and HbA_1c_: coronary artery calcium with SIF and CLF; intima-media thickness with SIF and LW-1; and left ventricular mass with LW-1 and CLF.

**Conclusions:**

LW-1 is a novel risk marker that is robustly and independently associated with the future progression of microvascular disease, intima-media thickness and left ventricular mass in type 1 diabetes.

*Trial registration* NCT00360815 and NCT00360893 at clinicaltrials.gov

**Electronic supplementary material:**

The online version of this article (doi:10.1186/s12933-016-0343-3) contains supplementary material, which is available to authorized users.

## Background

Advanced glycation end-products (AGEs) are a highly diverse group of amino-carbonyl compounds whose formation is initiated by the reaction of reducing sugars and oxoaldehydes with proteins. Glucose nonenzymatically reacts with free lysyl amino groups in proteins to form a Schiff base which rearranges to the more stable Amadori product [1]. The latter, in turn, undergoes a series of reactions to form insoluble, yellow, fluorescent and highly cross-linked products [2]. Protein-linked *long wavelength fluorescence* (*LWF*) ~ excitation (ex)/emission (em) 370/440 nm was introduced by us years ago as a surrogate marker for AGE formation [3] and has since been widely used in both clinical and experimental studies of diabetes [4]. In skin essentially most of the LWF is linked to the insoluble collagen fraction as “collagen-linked fluorescence” (CLF) which progressively increases with chronological age [2]. For CLF, insoluble collagen is prepared by extraction of skin and solubilized by enzymatic digestion to measure LWF [3]. Spectroscopically, diabetic samples show increased fluorescent intensity at 370/440 nm compared with healthy control subjects [2]. However, CLF is unable to differentiate between fluorophores with overlapping fluorescent spectra such as those derived from glycation *vs.* lipid peroxidation and other endogenous fluorophores [5–7].

In recent years, clinical interest in LWF as a marker of diabetic complications has rekindled with the development of diagnostic devices capable of noninvasively measuring LWF in human forearm skin [8, 9]. Referred as *skin autofluorescence* (SAF) [9] or *skin intrinsic fluorescence* (SIF) [8], these studies revealed strong correlations between non-invasively measured LWF in skin from individuals with type 1 and type 2 diabetes and the presence of micro- and macrovascular complications [10, 11] which have been strongly associated with AGE formation. However, besides collagen, other interstitial, cellular and vascular components can exhibit autofluorescence as well [12]. Additionally, the cause and effect relationship between the SAF/SIF readings and diabetic complications is not totally clear and there are numerous confounding factors such as sex, ethnicity and race on the interpretation of the fluorescent measures made at broadband wavelengths, ex/em 375–456/435–655 nm. Thus, there is a pragmatic need to understand the chemical nature of skin autofluoresence as to specific AGE structures which presently is poorly understood [13].

In view of the fact that the biochemical nature of LWF and the role of glycation vs. oxidation in its formation remains elusive [5, 14], we recently identified a major acid-labile fluorescent molecule with LWF in enzymatic digest of human insoluble skin collagen, named LW-1 [15]. In skin samples obtained at autopsy, levels were significantly elevated by age, diabetes, and end-stage renal disease. LW-1 has a molecular weight of 623 Da, a UV absorption maximum at ~348 nm and fluorescence maxima at ex/em 348/463 nm. Although its full chemical structure is still elusive, NMR analysis showed that it has a lysine residue in an aromatic ring coupled to a sugar molecule that is reminiscent of AGEs [15]. Because of these findings, we wanted here to determine the relationship between LW-1 vs. diabetes duration, glycemia and severity of complications in patients with type 1 diabetes using skin biopsy samples from the Diabetes Control and Complications Trial (DCCT) with long-term follow-up in the subsequent Epidemiology of Diabetes Interventions and Complications (EDIC) study. Furthermore, because these patients have up to a tenfold higher risk for the development of cardiovascular disease (CVD) compared with the general population [16–18], we additionally wanted to investigate the comparative relationship of the aforementioned markers of LWF in skin; i.e. CLF, SIF and LW-1, to various indices of subclinical CVD previously assessed in the DCCT/EDIC cohort. These include coronary artery calcification (CAC), carotid artery intima-media thickness (IMT) and various markers of myocardial dysfunction, particularly left ventricular hypertrophy/mass (LVM).

## Methods

### Study subjects

As previously described [19, 20], *two* 4 mm skin punch biopsies were obtained from the medial region of the right buttock from a total of 216 DCCT volunteers 1–2 years prior to DCCT closeout in 1993. Informed consent for biopsies was collected from volunteers [19]. The biopsies were stored under argon at −80 °C and processed as described in the recent publication [20]. Retinopathy, albumin excretion rate (AER), confirmed clinical neuropathy and HbA_1c_ were assessed at DCCT closeout as described in detail elsewhere [21]. In addition, consenting subjects were followed in EDIC and retinopathy was assessed at least once during EDIC years 13–16 [22] in each subject ≈ one-fourth subjects per year. Biopsies were also obtained from 42 healthy individuals between ages 21 and 50 years as age-matched controls [19].

### Biochemical standards

LW-1 was purified from an enzymatic digest of insoluble collagen extracted from a pool of human skin obtained by autopsy as previously described [15]. A standard was prepared based on published molecular data [15]. AGE standards including stable isotopes used as internal standards for fructosyl-lysine (i.e., Amadori product), carboxymethyl-lysine (CML), carboxyethyl-lysine (CEL), glyoxal hydroimidazolone (G-H1), methylglyoxal hydroimidazolone (MG-H1) and glucosepane have been described elsewhere [23].

### Processing of skin biopsies

The collagen digests used in this study were identical with those previously used for the determination of AGEs in the second skin punch biopsy [20]. Briefly, an insoluble delipidated pellet (mean recovery per biopsy was 7.2 mg; i.e., 92 %, range 72–100 %) was digested enzymatically and sequentially with collagenase, peptidase, pronase, and aminopeptidase as described [15] in order to release acid sensitive AGEs and LW-1 without destruction. Collagen content was determined in the digests by the hydroxyproline assay [24] assuming a content of 14 % hydroxyproline by weight [2].

### Measurement of LW-1 by HPLC monitored by fluorescence

Each digest ~100 µl was filtered using a 3 kDa molecular weight cut-off (MWCO) 0.5 ml filtering device (Amicon Ultracel UFC5003, Millipore Corp., Billerica, MA) centrifuged at 6000×*g* for ≈45–60 min (MRX-151 TOMY). An aliquot 14 µl of each digest (~66 ± 9 µg collagen) was injected onto a 15 cm × 2.1 mm, 3 µm Discovery HS C18 column preceded by a 2 cm × 2.1 mm guard column same (Supelco-Sigma, St. Louis, MO). The HPLC, solvents and gradient program were previously described by us [15]. Eluate from the column flowed onto a JASCO Model 821-FP fluorescence detector monitored at ex/em 348/463 nm at gain 10×. Each chromatogram was integrated for the LW-1 peak area relative to that of the homemade LW-1 standard (Additional file 1) and the results were expressed as pmoles LW-1 per mg collagen measured by hydroxyproline assay in the acid hydrolysate of each digest.

### Calculation of fluorescence recovery due to LW-1

Fluorescence recovery attributable to LW-1 compared to CLF at ex/em 348/463 nm was determined by manually collecting the LW-1 peak eluting from the HPLC and comparing its fluorescence yield with that of the digest itself at wavelengths same. An enzyme blank was also injected into the HPLC and the eluate with the same retention time as LW-1 (i.e., background) was also collected and subsequently subtracted from the fluorescence of the LW-1 peak. HPLC eluates were reconstituted to 2 ml with HPLC solvent and fluorescence was determined with a JASCO 821-FP detector. Percent fluorescence recovery for LW-1 was calculated as [(peak LW1–peak enzyme blank)/(injected collagen digest – enzyme blank)]*100.

### Quantitative analysis of AGEs by liquid chromatography-tandem mass spectrometry (LC-MS/MS)

Fructosyl-lysine, glucosepane, CML, CEL, G-H1, MG-H1 were assayed by liquid chromatography—tandem mass spectrometry (LC-MS/MS) using isotope dilution technique as recently published by us [20]. In short, each digest was filtered (as described above) followed by injecting ~40 µl onto a Waters 2695 Alliance HPLC system using two Hypercarb columns (ThermoFisher) connected in series. Separations were made using chromatographic methods described by Thornalley et al. [25]. The eluate was directed to a Micromass Quattro Ultima mass spectrometer (Waters) which monitors for both the molecular and product ion fragments of each AGE assayed [23]. For this work, the values were obtained from our recent paper [20]. Pentosidine and collagen solubility values were taken from the 1999 study [19].

### Quality controls (QCs)

Since a large number of digests were injected onto the HPLC over the course of 6 weeks, assays were monitored for quality assurance by a Levey-Jennings plot (Additional file 2). For LW-1, the low and high quality controls (i.e., LQC and HQC) consisted of injecting standardized digests prepared from skin samples obtained at autopsy from a 22 and 38 year-old patient, respectively. Aliquots of LQC and HQC digests ~64 µg collagen in 10 µl were injected onto the HPLC approximately every 2–4 days while LW-1 used as a quality control (QC) standard was injected every day (~3 pmol in 5 µl).

### Measurement of skin intrinsic fluorescence (SIF)

SIF was determined noninvasively on the left volar forearm skin of patients at EDIC years 16–17 using the SCOUT DS instrument (Miraculins, Inc., Winnipeg, Manitoba, Canada) according to procedures detailed elsewhere [18, 26]. For these analyses, the excitation (ex) was centered at 375 nm using a light-emitting diode (LED) followed by measuring fluorescence detected over the broadband emission (em) range of 435–655 nm. Measurement of skin reflectance (R) was used to compensate for absorbance due to melanin and hemoglobin as well as subject-specific light scattering; i.e., individual differences due to the presence of wrinkles and hair follicles as well as dermal collagen concentration and organization [10]. An intrinsic fluorescence correction formula was used as follows: f_ex.em_ = F_ex.em_/$(\text{R}_{\text{ex}}^{\text{k}_{\text{ex}}} \text{R}_{\text{em}}^{\text{k}_{\text{em}}})$ where the measured fluorescence, F_ex.em_, is divided by reflectance values measured over both the excitation (R_ex_) and emission (R_em_) regions. The reflectance values are adjusted by the dimensionless exponents, k_ex_ and k_em_, set to 0.6 and 0.2, respectively. The resulting intrinsic fluorescence f_ex.em_ was integrated over the 435–655 nm spectral region and multiplied by 1000 to represent SIF, reported in arbitrary units. The values used for k_ex_ and k_em_ were previously determined in the Pittsburgh Epidemiology of Diabetes Complications (EDC) study to be relevant for the 375 nm excitation which had the strongest association with diabetes-related complications in the Pittsburgh EDC cohort [27]. In the present study, the *first* SIF measurement per subject was used for all plots and analyses [18, 26].

The specificity of the SCOUT for fasting plasma glucose (FPG) was previously reported to be 77 % [10]. Likewise, the sensitivity of this instrument for FPG, HbA_1C_, and SIF (as contributable to dermal AGEs) was previously determined to be 58, 64 and 75 %, respectively [10].

The general technical requirements for the suitable noninvasive estimation of the diabetic levels using a skin fluorescence spectrometer have previously been published [10, 28, 29]. For noninvasive diabetes screening using the SCOUT, statistical analysis involving both principal component and linear discriminant analyses were applied to SIF spectra to differentiate whether an individual belonged to the diabetic vs. nondiabetic group [10].

### Cardiovascular disease (CVD) outcomes

Coronary artery calcification (CAC) was determined by computed tomography (CT) at EDIC years 7–9 by methods previously described [17]. Intima-media thickness (IMT) of the common carotid artery was measured by ultrasonography and image analysis at EDIC years 6 and 12 according to procedures detailed elsewhere [30]. Left ventricular mass (LVM), volumes, and functional parameters were determined from short axis cine images covering the heart from base to apex throughout the cardiac cycle using cardiac magnetic resonance imaging (cMRI) at EDIC years 14–16 by methods previously described [31].

### Statistics

The precision of the LW-1 assay was determined by intra-day and inter-day coefficients of variability (% CV). The coefficient of variability (CV) was calculated as follows: % CV = (mean level/SD)*100 where SD: standard deviation. Intra-day % CV was determined by repeated injections of the LW-1 standard on the same day over the 6 week course of the assay. Inter-day % CV was determined by repeated injections of the LQC, HQC and the LW-1 QCs made on different days over the assay period for time points shown on the Levey-Jennings plot (Additional file 2). Linear regression analyses including computation of the regression line and its 95 % confidence intervals (CI) of prediction were done using SigmaPlot 11.0 software (Systat Software, Inc., San Jose, CA). The comparisons among LW-1, CLF and SIF were made by partial correlation analyses using SPSS v.11.5 software (IBM SPSS, Chicago, IL). All other statistical methods have been detailed elsewhere [19, 20] including Spearman correlation, linear regression models, multivariate logistic regression models, entropy R^2^, odds ratio and adjustment of multiple tests.

## Results

### Chromatographic analysis of LW-1

Using the stated gradient method, LW-1 eluted at ≈86 min in the chromatograms (Additional file 1). A Levey-Jennings plot was constructed to monitor instrument performance over time indicated no drift requiring an adjustment of the data (Additional file 2). Thus, the data were used “as is”. This analysis revealed an intra-day CV of <4 % for the LW1 QC standard and an inter-day CV of <4, 8 and 10 % for the high (HQC), low (LQC) and LW-1 QC standard, respectively. Of note is that LW-1 is the single major fluorescent peak in samples from both DCCT and control donors (Additional file 1).

### LW-1 correlation with age, diabetes duration and glycemic control

**Fig. 1:**
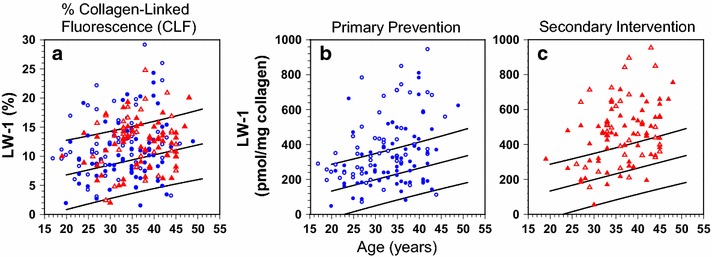
Unadjusted LW-1 vs. age in skin collagen digests of DCCT patients. For all graphs, regression lines and 95 % CI prediction for nondiabetic controls are shown. **a** Unadjusted LW-1 expressed as a percentage of total fluorescence at ex/em 348/463 nm in skin collagen digests vs. age. Percent LW-1 was determined by manually collecting the LW-1 peak by HPLC and comparing its fluorescence yield with that of the digest itself at wavelengths same as described in the “Methods” section. **b** and **c** Unadjusted LW-1 levels vs. age at DCCT closeout in the primary and secondary cohorts. Regression lines: **a** y = 3.4 + 0.172x, r = 0.48, P = 0.002, n = 39. **b** and **c** y = 1.8 + 6.6x, r = 0.64, P < 0.0001, n = 42. Treatment (cohort) *circle*, conventional (primary); *triangle*, conventional (secondary); *filled circle*, intensive (primary); *filled triangle*, intensive (secondary)

**Fig. 2:**
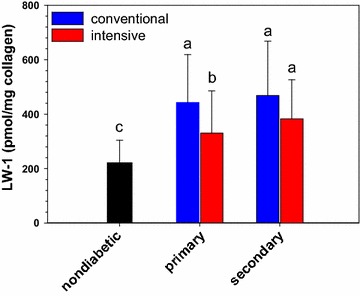
The effect of intensive vs. conventional glycemic therapy on LW-1 levels after adjustment for age and diabetes duration in the primary and secondary DCCT cohorts at DCCT closeout. LW-1 responds significantly to lowering of glycemia after adjusting for age and duration of diabetes (nondiabetic controls: age-adjusted). Each *bar* represents the mean ± SD. Means with a different letter superscript are significantly different (P ≤ 0.0001)

LW-1 significantly (P < 0.0001) increased with age in the control individuals without diabetes as well as in the DCCT patients treated conventionally and intensively for glycemic control (Additional file 3). For this latter group, LW-1 responded well to intensive therapy in the primary prevention cohort (no retinopathy at baseline) but less well in the secondary intervention cohort (mild retinopathy at baseline) (Fig. 1b, c; Fig. 2). In the primary cohort, levels were significantly (P < 0.0001) lowered by intensive vs. conventional therapy with many of these data points *within* the 95 % CI determined for the controls (Fig. 1b). In comparison, in the secondary cohort, intensive therapy lowered LW-1 in some patients, but less in others relative to conventional therapy, and many of the intensive group data points remained *outside* the 95 % CI determined for the controls (Fig. 1c). Overall, the effect of intensive therapy was still significant (P = 0.037) in this cohort.

Spearman correlation analysis across DCCT treatments showed that levels were significantly (P < 0.0001) elevated by both age and diabetes duration (data not shown). After adjustment for these two factors, analysis reconfirmed that LW-1 levels were significantly (P = 0.0001) attenuated by intensive vs. conventional glycemic treatment (Fig. 2). However, mean levels remained significantly (P < 0.0001) elevated in patients undergoing intensive therapy *vs.* the controls without diabetes (Fig. 2).

### Correlation with AGEs

Regression analysis was used to explore the relationship between LW-1 and other collagen markers measured by our laboratory in the DCCT. AGE markers including percent insolubility, CLF, fructosyl-lysine, glucosepane, pentosidine, CML, MG-H1 and CEL all correlated (r = 0.15–0.70) significantly (P < 0.02) with LW-1 (Additional file 4). Furthermore, except for CEL, the statistical significance for all these relationships persisted after LW-1 was adjusted for age and diabetes duration in DCCT participants (Additional file 5). Interestingly, LW-1 most strongly correlated with glucosepane: r = 0.6–0.7, P < 0.0001 (Additional file 4, Additional file 5) suggesting that glucose may be part of the LW-1 structure (see "Discussion" section).

### Correlation of LW-1 with Collagen-Linked Fluorescence (CLF) and noninvasive Skin Intrinsic Fluorescence (SIF)

**Fig. 3:**
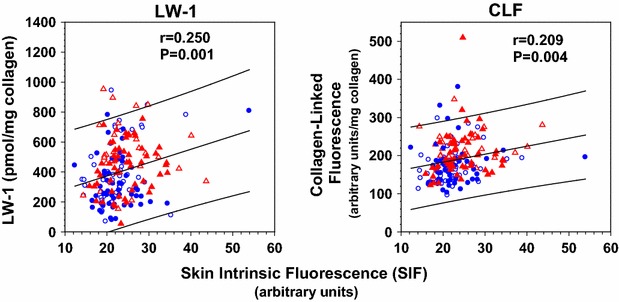
Plots of unadjusted specific intrinsic fluorescence (SIF by SCOUT DS) vs. unadjusted LW-1 (*left*) and unadjusted collagen-linked fluorescence/CLF (*right*) in DCCT/EDIC participants (see “Methods”). LW-1 and CLF were assayed in enzymatic digests of insoluble collagen prepared from skin biopsies obtained from the buttock region of participants at DCCT closeout (1993). SIF was obtained by noninvasive measurements of autofluorescence made by the SCOUT DS on the underside of the left forearm skin near the elbow for most DCCT participants at EDIC years 16–17 (2009–2010). Regression line and 95 % CI prediction: CLF y = 141 + 2.1x, r = 0.209, P = 0.004, n = 185; LW-1: y = 195 + 8.9x, r = 0.25, P = 0.001, n = 185. Symbols used: see Fig. 1

LW-1 accounted for up to ~30 % of CLF levels measured in chromatograms at ex/em 348/463 nm; i.e., the fluorescence maxima of LW-1 (Fig. 1a). As similar to the analysis in the above, LW-1 increased in DCCT patients vs. the controls (P = 0.003) and decreased with intensive vs. conventional therapy (P = 0.005, Fig. 1a). The association with noninvasive SIF measured by SCOUT DS was positive and strongly significant even though SIF was determined 16–17 years *after* the skin biopsy used to measure LW-1 (Fig. 3). SIF significantly correlated with LW-1 both before (P = 0.001, Fig. 3) and after age correction (P = 0.046, data not shown). In contrast, SIF significantly correlated with CLF before age correction (P = 0.004, Fig. 3), but not after (P = 0.16, data not shown). In further analysis, LW-1 significantly correlated with CLF (P < 0.0001), but not SIF (P = 0.084), after further correction for diabetes duration (Additional file 5). Thus, LW-1 and CLF were more closely associated in this study due to measurements made within the same buttock skin biopsy (1993). In turn, unsurprisingly, these two measures were considerably less associated with SIF which was noninvasively measured on volar forearm skin many years later (2009–2010).

### Correlation with indices of cumulative glycemia (HbA_1c_)

LW-1 was strongly associated with recent measures of glycemia such as at the time of biopsy (R^2^ = 10.1, P < 0.0001) or the year preceding the biopsy (R^2^ = 9.6, P < 0.0001) and significantly correlated with mean HbA_1c_ ~7–10 years prior to the biopsy (R^2^ = 9.9, P < 0.0001). As expected, it correlated less with glycemia at initial screening (R^2^ = 2.7, P < 0.015). Subsequently, ≈10 % of variation in LW-1 levels was explained by HbA_1c_ (Additional file 6).

### Correlation with past and future progression of microvascular complications

**Fig. 4:**
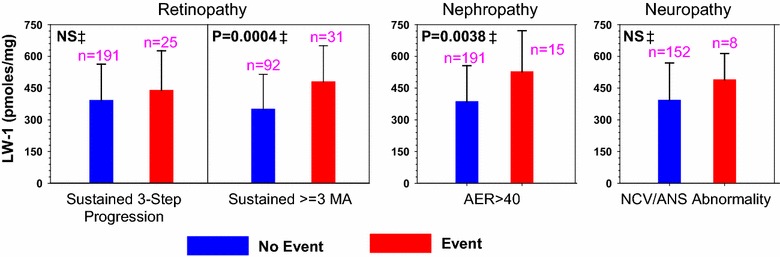
Univariate regression models of complications vs. age- and duration-adjusted LW-1 levels (mean ± SD) across treatment groups at DCCT closeout. *MA* microaneurysms, *AER* albumin excretion rates, *NCV/ANS* nerve conduction velocity/autonomic nervous system, *NS* nonsignificant (P > 0.05). Analysis was limited to those with no respective complication at DCCT baseline (excluding 10 subjects with AER > 40, 19 with confirmed clinical neuropathy, and the secondary cohort for sustained ≥3 MA). Indices of retinopathy, microaneurysms, nephropathy and neuropathy were quantified as previously described [19]. ‡ P value is from a Likelihood Ratio test (LRT) between the respective reduced model and the full model

LW-1 was evaluated with respect to patient complications which progressed over the DCCT, but limited to patients with no respective complication at DCCT baseline with exclusions (see Fig. 4). Complications included retinopathy, nephropathy and neuropathy (Fig. 4) with clinical outcomes defined according to DCCT criteria [19, 20, 32] as either sustained 3-step progression in retinopathy, sustained ≥3 microaneurysms (retinopathy), albumin excretion rates >40 mg/24 h (nephropathy), or motor nerve abnormalities (neuropathy). In these evaluations, univariate regression analyses were used to model type of complication vs. LW-1 levels after adjustment for age and diabetes duration averaged across all treatments and cohorts (Fig. 4). The results showed that mean levels of LW-1 were elevated in patients for all complications listed in Fig. 4 with significant associations with microaneurysms (P = 0.0004) and AER (P = 0.0038). Similarly, at EDIC years 13–16, age and duration-adjusted LW-1 levels were significantly (P = 0.023) associated with 3-step progression of retinopathy (Table 1). Following adjustment for HbA_1c_ up to the time of biopsy; i.e., mean HbA_1c_ during the DCCT, AER was the only complication that remained significant (P = 0.036, Table 1).

**Table 1:** Logistic regression models of microvascular complications vs. age- and duration-adjusted LW-1 levels with and without adjustment for DCCT mean HbA1c up to the biopsy across treatment groups

Outcome	Total N	Event N	LW-1^a^			LW-1 Adjusted for HbA1c ^b^			DCCT Mean HbA1c^a^		DCCT Mean HbA1c Adjusted for LW-1^b^	
			Odds ratio^c^	Entropy R2^d^	P value^e^	Odds ratio^c^	Entropy R2^d^	P value^e^	Entropy R2^d^	P value^e^	Entropy R2^d^	P value^e^
DCCT complications (at DCCT closeout*)*												
Retinopathy/microaneurysms (sustained ≥3 MA ever in DCCT) (primary cohort)	123	31	1.97 (1.40, 3.27)	9.0	*0.0004*	1.66 (1.00, 2.33)	2.3	0.077 (NS)	14.9	*<0.0001*	8.1	*0.0008*
Nephropathy (AER closest to biopsy > 40 mg/24 h)	206	15	1.97 (1.19, 3.27)	7.8	*0.0038*	1.66 (1.00, 2.76)	4.1	*0.0357*	8.3	*0.0028*	4.6	*0.0259*
EDIC complications (EDIC years 13–16)												
Retinopathy/progression (3 or more step progression)	213	91	1.37 (1.04, 1.81)	1.8	*0.023*	1.09 (0.80, 1.48)	0.1	0.58 (NS)	10.5	*<0.0001*	8.8	*<0.0001*

Abbreviations used: see Fig. 4. Analysis limited to those with no respective complication at DCCT baseline excluding 10 subjects with AER >40 mg/24 h and the secondary cohort for sustained ≥3 MA

^a^Unadjusted models are from univariate logistic regression with LW-1 or DCCT mean HbA1c up to the biopsy time as the risk factor

^b^Adjusted models are from logistic regressions with both LW-1 and the DCCT mean HbA1c up to the biopsy time as risk factors

^c^Odds ratio is calculated based on one SD increase in LW-1; i.e., 170

^d^Entropy R2 is calculated as the ratio of Chi square from a likelihood ratio test and −2*log transformation of the likelihood from the null model with intercept only

^e^P value is from the Likelihood Ratio Test (LRT) between the respective reduced vs. full model

### Correlation of LW-1 with subclinical macrovascular complication progression compared with CLF and noninvasive SIF

**Fig. 5:**
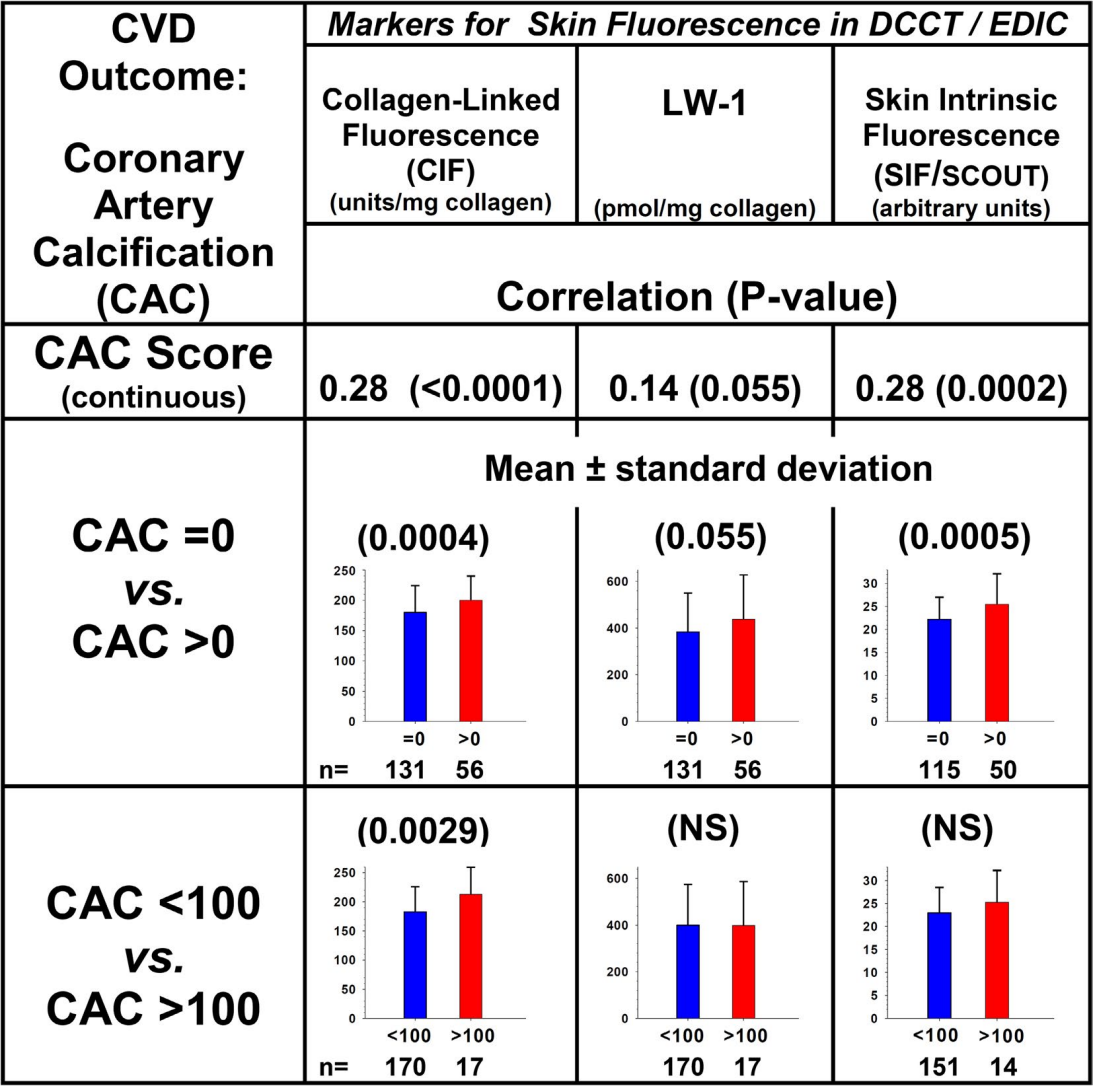
Spearman correlations for markers of skin fluorescence vs. coronary artery calcification (CAC). Markers were adjusted for age and diabetes duration. CAC was determined at EDIC years 7–9 by computed tomography as scores in Agatson units

Spearman correlation analysis (univariate) was used to compare all three currently known fluorescent markers for their associations with subclinical cardiovascular disease progression. The results show that LW-1 was borderline nonsignificantly (P = 0.055) associated with CAC; i.e., continuous CAC score and CAC score >0 (Fig. 5). However, these associations were much stronger for CLF (P < 0.0001) and SIF (P = 0.0002) whereby CLF was the only parameter that was significantly associated with the severity of CAC score; i.e., CAC = 0 vs. CAC > 0 (P = 0.0004) or CAC < 100 vs. CAC > 100 (P = 0.003). Both LW-1 and CLF were weakly (P < 0.05) associated with IMT change at EDIC year 6 and 12, respectively. In comparison, SIF was significantly (P < 0.01) associated with IMT progression at both 6 and 12 years (data not shown). Finally, left ventricular mass (LVM) was selectively associated with LW-1 (P = 0.011) and CLF (P = 0.0075) while SIF was associated (P < 0.01) with ejection fraction (data not shown).

**Fig. 6:**
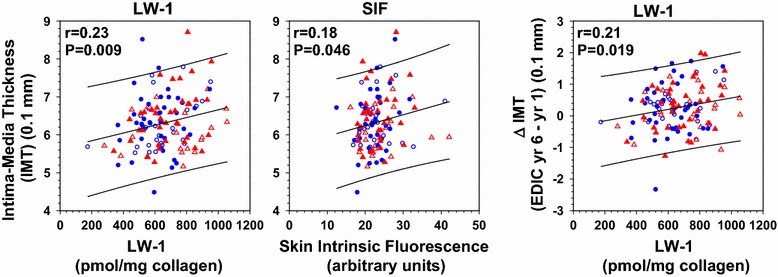
Linear plots of intima-media thickness (IMT) as well as the change in IMT between EDIC years 1–6 vs. markers for skin fluorescence (LW-1, SIF), as indicated. Preliminary Spearman correlation analysis showed that LW-1 and SIF (but not CLF) significantly (P < 0.05) correlated with IMT at EDIC year 6, but not at year 1. Further linear regression analysis showed that IMT (for both years) significantly correlated with age (P < 0.0001), HbA_1c_ (P = 0.013) and borderline nonsignificantly with diabetes duration (P = 0.068). Thus, all variables in these graphs have been adjusted for age, diabetes duration and HbA_1c_ (both DCCT and EDIC). For all graphs, the regression line and 95 % CI prediction of data points are shown: (*left*) IMT = 5.64 + 0.001 (LW-1), n = 127; (*middle*) IMT = 5.68 + 0.028 (SIF), n = 121; (*right*) change in IMT between EDIC year 1 and 6, ∆IMT = 0.001(LW-1)–0.339, n = 121. All values for IMT and ∆IMT have been multiplied by 10. For each graph, the correlation (*r*) and significance (*P*) are inserted. Symbols used: see Fig. 1

**Fig. 7:**
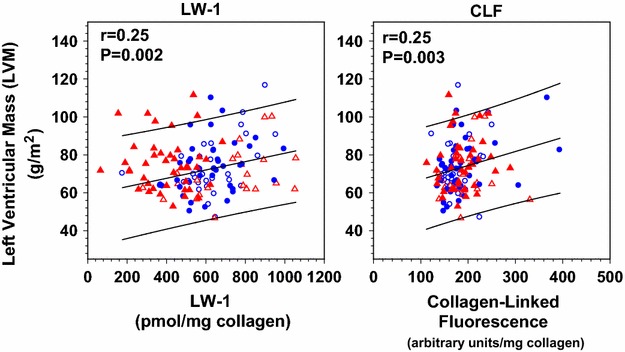
Linear plots of left ventricular mass (LVM) at EDIC years 14–16 vs. LW-1 and CLF, as indicated. Preliminary Spearman correlation analysis showed that both LW-1 and CLF were significantly (P < 0.05) associated with LVM, but not SIF (P > 0.05). LW-1 and CLF were adjusted for age, diabetes duration and HbA1c (both DCCT and EDIC). However, LVM did not significantly correlate with any of these latter variables, thus no adjustments were made (P = 0.27, P = 0.98, P > 0.25, respectively). For both graphs (n = 142), the regression line and 95 % CI prediction are shown: (*left*) LVM = 59 + 0.021 (LW-1); (*right*) LVM = 59 + 0.081 (CLF). For each graph, the correlation (*r*) and significance (*P*) are inserted. Symbols used: see Fig. 1

Multivariate regression analyses were performed to investigate the effects of adjustment for age, diabetes duration and especially HbA_1c_ during the DCCT and EDIC phase of the study. In short, these analyses showed that CAC was significantly associated with CLF (P ≤ 0.021) and SIF (P ≤ 0.046); IMT with LW-1 (P ≤ 0.02) and SIF (P < 0.01); and LVM with LW-1 (P ≤ 0.008) and CLF (P ≤ 0.035). Subsequently, linear plots were made of these significant associations which are shown in Figs. 6 and 7 for IMT and LVM, respectively.

## Discussion

### Issues using autofluorescence/LWF as a marker for accumulation of AGEs

LWF has traditionally been used as a marker and measure for AGE modifications in skin studies both *in vivo* [12, 33] and *in vitro*; e.g., reconstructed skin model for aging of human skin [34]. A number of issues have arisen in the assessment of AGEs by LWF which need to be considered in the interpretation of the results. First, as previously mentioned, LWF is structurally poorly defined in that other molecules besides AGEs may fluoresce and contribute to measured levels. Undoubtedly, however, as presented in another paper by us involving the same DCCT/EDIC patients [35], many important AGEs are *not* fluorescent and significantly increase with the progression of diabetes and CVD. Secondly, LWF is significantly and highly influenced by chronological age. For example, in a study of obesity in healthy individuals, LWF measured by the AGE Reader as SAF significantly increased in the skin of obese compared with non-obese subjects; i.e., attributable to accelerated AGE formation, but the statistical difference between the two cohorts was abolished after the correction for age and smoking [12]. Likewise, in another study, both CLF and SAF were significantly and positively correlated with pulse wave velocity (PWV) in association with vascular stiffness and function in patients with coronary heart disease [33]. However, no adjustment was made for the covariance of age among these variables, thus making the interpretation of the relationship between LWF and PWV obscured. Thirdly, the relationship between LWF and glycemia has not always been definitive, but definitely more correlative for hyperglycemic *vs.* normoglycemic conditions. For example, in the obesity study just mentioned by den Engelsen [12], there was no association between SAF and fasting blood glucose even though the obese individuals had significantly (P < 0.001) higher blood glucose levels *vs.* the non-obese (mean 90 ± 18 vs. 86 ± 11 mg/dl, respectively). Similarly, in the coronary heart disease study by Hofmann et al. [33], there was no association between CLF and blood glucose (114 ± 36 mg/dl) or HbA_1c_ (6.3 ± 0.7 %). However, this study consisted of a small number of patients (n = 52) with and without type 2 diabetes (n = 19). In a large clinical trial involving n = 1185 DCCT/EDIC patients with type 1 diabetes, SIF positively and significantly correlated (r = 0.26, P < 0.0001) with 25 year mean HbA_1c_ (overall mean 8.0 ± 1 %, range ≈5–12 %) whereby the correlation got even stronger (r = 0.46) after SIF was adjusted for a host of factors including age, smoking, skin tone, glomerular filtration rate and clinic latitude [18].

### The relationship of LW-1 with age and glycemic therapy in the treatment of type 1 diabetes

The above results show that LW-1 levels increased with age in insoluble skin collagen from both DCCT and control individuals and its formation was further catalyzed by type 1 diabetes. LW-1 levels measured in this tissue near DCCT closeout were significantly reduced after glucose levels were lowered by intensive therapy over the 9 year period of the DCCT. This effect was most pronounced in the primary prevention cohort and less so in the secondary intervention cohort. Not surprisingly, similar results were previously observed for other markers measured by our laboratory [19, 20, 35] since the secondary cohort had longer duration of diabetes and the presence of complications at baseline *vs.* the primary cohort with shorter duration of diabetes and no complications at baseline. In other words, LW-1 formation is more easily prevented, or even reversed the younger the age and the less advanced the disease are.

### The relationship of LW-1 with glycemia and diabetic complications

Our previous studies on the relationship between skin collagen markers and glycemia revealed associations with past cumulative glycemia that varied in strength depending upon the analyte. For example, glucosepane was strongly associated with mean glycemia at all time points during the DCCT from baseline on [20]. Here we found that LW-1 correlates with both recent and long-term glycemia, explaining up ≈10 % of the variability in HbA_1c_ levels. This suggests that LW-1 may be potentially valuable for the assessment of diabetic complications related to hyperglycemia.

In comparison, in the analysis of LW-1 association with *microvascular* complications, levels adjusted for age and duration increased in all diabetic complication outcomes studied, although statistical significance was reached only for microaneurysms and AER. At follow-up in EDIC, adjusted levels were significantly associated with 3-step progression of retinopathy 13–16 years following DCCT closeout. However, in all these analyses, except for AER (P = 0.036), the associations were weakened by further adjustment for HbA_1c_ perhaps suggesting that LW-1 has a more important role as a marker rather than a culprit of *microvascular* disease. These results also suggests that the predictive effect of LW-1 on microvascular complications is to some extent mediated by glycemia and that LW-1 may be more proximal to the causal mechanism by which HbA_1c_ affects outcomes. In this regard, our recent study showed that several AGEs were much more strongly associated with microvascular disease progression in spite of adjustment for mean glycemia than LW-1 [32]. The order for retinopathy was (strongest to weaker) glucosepane > fructose-lysine (furosine) > other AGEs; for nephropathy, fructose-lysine (furosine) > other AGEs; and for neuropathy, methylglyoxal hydroimidazolone (MG-H1) > fructose-lysine (furosine) > other AGEs.

Concerning the *macrovascular* complications, LW-1 was found most significantly related to carotid IMT and cardiac LVM as outcome markers for atherosclerosis and cardiac hypertrophy/dysfunction, respectively. Importantly, unlike the microvascular outcomes evaluated above, statistical significance for IMT and LVM remained significant *even* after adjustment for HbA_1c_ levels (Figs. 6, 7). These observations may be related to our previous finding that LW-1 levels are highly elevated in patients with renal failure, especially those with end-stage renal disease undergoing dialysis [15]. The latter is well known to greatly accelerate CVD outcomes including atherosclerosis [36] and heart hypertrophy [37]. Conversely, none of the DCCT/EDIC participants in this study were in renal failure and accordingly LW-1 was not associated with CAC outcome as a marker for atherosclerosis.

### The relationship of SAF/SIF measured by skin readers with diabetic complications

Numerous studies with the skin readers have shown that autofluorescence correlated strongly with the development of nephropathy [38] and neuropathy [39] including its association with the development of diabetic foot ulcers [40]. Conversely, its association with retinopathy was not nearly as strong. Its statistical significance was highly dependent upon whether levels were corrected for confounding risk factors of diabetic complications; namely, age, duration of diabetes and HbA_1c_. Subsequently, the relationship between SAF with the development of retinopathy was found significant after adjustment for age [41], but not after adjustment for duration [41] and/or HbA_1c_ [9]. Furthermore, in a prospective study with a follow-up period of over 3 years involving 967 patients with type 2 diabetes, no significant relationship between SAF and retinopathy was found [42].

### The relationship of CLF with glycemia and diabetic complications

Previously, we showed that skin CLF at ex/em 370/440 nm accounted for up to ≈7 % of the variation in HbA_1c_ levels at DCCT closeout [19] vs. 10 % for LW-1 in the present study. After adjustment for age and duration, CLF was significantly associated with nephropathy and neuropathy, but not retinopathy [19]. Prospectively, age- and duration-adjusted CLF determined at DCCT closeout significantly increased in relationship with the progression of nephropathy (P = 0.0033) and retinopathy (P = 0.0008) at EDIC years 9–10, but the statistical significance was nullified by the further adjustment of levels for HbA_1c_ [43]. Thus, CLF is not a robust marker for microvascular disease compared with fructose-lysine (furosine), glucosepane and MG-H1 as discussed above.

### The significance of LW-1 as a marker for diabetic complications in comparison with other fluorescent and non-fluorescent markers

The significance of LW-1 as a marker, or possible culprit of complications, depends on two conditions; i.e., its biochemical origin and its ability to possibly predict complication progression better than other fluorescent and non-fluorescent AGEs. Concerning the first condition, its biochemical origin is still under investigation. The second issue is in part the topic of this study and is closely related to commercially-introduced diagnostic devices that measure autofluorescence of human forearm skin using broadband fluorescence technology. Together, these previous studies revealed that autofluorescence measured by the skin readers was strongly associated with age, renal failure and diabetes [44, 45]. For diabetes, autofluorescence levels were associated with both micro- and macrovascular complications, cardiovascular mortality, and all-cause mortality [46] [reviewed by Bos et al. [47] ]. In type 1 diabetes, these levels were directly correlated with coronary artery disease [38] and CAC [27], and inversely correlated with elasticity of both small and large arteries [11]. Additionally, these levels also increased in subjects with artery stenosis and peripheral artery disease [48].

Questions persist as to whether LW-1 is better than other markers in terms of its significance and detectability in vivo; i.e., its ability to be technically separated from other AGE fluorescence. This first question has been addressed in the correlative statistical analyses of subclinical endpoints with fluorescent markers. Using Spearman correlations and the recently published data on non-fluorescent AGEs [35], the order of significance for IMT was LW-1 > pepsin soluble collagen > fructose-lysine > glucosepane > CLF (all P < 0.05). Except for fructose-lysine (furosine), none of the traditional AGEs predicted CAC while LW-1 was only borderline associated with it (P = 0.055). LW-1 was the strongest predictor of left ventricular mass together with CML (P = 0.01) followed by pepsin soluble collagen and MG-H1 (P < 0.05).

In regard to the second question, deconvolution analysis of total fluorescence at ex/em 370/440 nm is certainly feasible and might be helpful for documenting the complexity of the compounds contributing to total fluorescence. However, limited structural information is expected from such analysis which is a major reason why we have not so far attempted to deconvolute CLF into its subcomponents.

### The strong correlation of LW-1 with glucosepane towards the putative structure of LW-1

LW-1 significantly correlated with other markers measured by our laboratory both before and after adjustment for age and diabetes duration. The strongest association was with glucosepane suggesting glucose may be part of the LW-1 structure. Yet, the biochemical relationship between LW-1 and glycation is still unclear. LW-1 is a highly fluorescent lysine-derived amino acid with a molecular weight of 623 Da, a UV maximum at 348 nm and fluorescent maxima at (ex/em) 348/463 nm due to an aromatic ring [15]. This agrees with our HSQC (heteronuclear single quantum coherence) 2D-NMR data which shows signals indicative of a 6-carbon sugar [15]. Finally, LW-1 is a major collagen modification with levels approaching those of glucosepane, the single major AGE and collagen crosslink in diabetic tissues. At this point however, a complete structure is not available yet and further work is needed to fully resolve its structure.

### Summary of the relationship between LW-1, CLF and SIF

The interrelationship between the three types of fluorescence referenced in this study can be summarized as follows: The specific LW-1 fluorophore contributes up to 30 % and perhaps more of total skin CLF measured at ex/em 348/363 nm. On the other hand, both CLF and LW-1 correlate strongly with autofluorescence (AF) (r = 0.2) [49]. However, CLF presumably also comprises other yet unidentified types of fluorophores that may not necessarily be associated with diabetic complications. Indeed, while AF/SIF are associated with severity of complications, two recent genome-wide association studies (GWAS) mention loci [50, 51] or coffee consumption [52] as being associated with skin fluorescence and type 1 diabetes, but not diabetic complications [50, 51].

## Conclusion

A major conclusion from the above studies is that LW-1 is an important long-term risk factor for the development of IMT and LVM associated with macrovascular disease. This risk factor remained significant after further adjustment for HbA_1c_. Vice versa, HbA_1c_ was not significantly associated with either IMT or LVM. Interestingly, while glucose-derived AGEs such as fructose-lysine/furosine and glucosepane are very strongly associated with microvascular disease [20, 32], these same markers are superseded by the fluorescent species in their association with progression of subclinical macrovascular disease. All three fluorescence markers reported here show some degree of correlation among each other, yet progress in structure elucidation of LW-1 and a precise understanding of the molecular basis of skin autofluorescence will be needed to understand their role in the elusive mechanistic link between hyperglycemia and the acceleration of macrovascular disease in diabetes.

## Additional files

10.1186/s12933-016-0343-3 HPLC-fluorescence chromatograms of LW-1 eluting from a 15 cm X 2.1 mm, 3 μm Discovery HS C18 column. For the standard, LW-1 ~ 6 pmoles was injected in a 10 μl volume. For DCCT samples, including the nondiabetic, digests of insoluble collagen prepared from skin biopsies were injected onto the HPLC in 14 μl volumes, each containing ~ 61 to 69 μg collagen. The low and high quality controls (LQC and HQC) consists of collagen digests prepared from autopsied skin samples from a 22 and 38 year-old patient, respectively (see Methods). The former died from meningitis while the latter died from ESRD secondary to type 1 diabetes. Totals of 64 μg collagen in 10 μl were injected onto the HPLC column. Peaks eluting > 100 minutes represent column washing and equilibration (see "Methods" section).
10.1186/s12933-016-0343-3 Levey-Jennings plot for LW-1 assay in the DCCT study. The plot consists of injection number on the x-axis (i.e., from 1 to 357 in consecutive order over the six week course of the HPLC assay) *vs*. integrated peak areas on the y-axis (relative fluorescence at ex/em 348/463 nm) for all samples injected (n = 216 DCCT + n = 42 nondiabetic controls + n = 13 repeats), LQC (n = 13), HQC (n = 14), LW-1 QC standard (n = 49) and enzyme blanks (n = 10).
10.1186/s12933-016-0343-3 LW-1 levels increase with age in insoluble skin collagen of DCCT participants at DCCT closeout (x: age, y: LW-1). *Controls*: subjects without diabetes, regression line and 95 % CI prediction, y = 0.05 + 7x, r = 0.64, P < 0.0001, n = 42. *Conventional*: regression line (dash line) y = 21 + 13x, r = 0.43, P < 0.0001, n = 94.* Intensive*: regression line (dash line) y = 11x - 22, r = 0.42, P < 0.0001, n = 122. The regression line and 95 % CI for the controls have been reproduced in the latter two graphs for comparison. Symbols used: treatment (cohort): *squares*, controls; *circles*, conventional (primary); *triangle*, conventional (secondary);* filled circle*, intensive (primary); *filled triangle*, intensive (secondary) 
10.1186/s12933-016-0343-3 Plots of unadjusted LW-1 levels vs. AGEs measured in insoluble skin collagen in the combined dataset: i.e., DCCT (n = 216) plus nondiabetic controls (n = 42). Levels of all measured parameters except collagen insolubility (%) and CLF (arbitrary fluorescence units/mg collagen) are stated in picomoles per mg collagen. For each graph, the linear regression line and 95 % CI prediction determined for the combined dataset are shown along with its correlation coefficient and statistical significance (P value). Symbols used: see Additonal file 3.
10.1186/s12933-016-0343-3 Partial correlation analysis of LW-1 with other skin collagen markers controlling for age and duration of diabetes.
10.1186/s12933-016-0343-3 LW-1 correlates better with recent rather than long-term cumulative glycemia. Univariate regression models of age- and duration-adjusted LW-1 (biopsy) *vs.* HbA1c at different sampling times in the DCCT.

## Abbreviations

AER: albumin excretion rate
AF: autofluorescence
AGEs: advanced glycation end-products
CAC: coronary artery calcification
EDC: Epidemiology of Diabetes Complications
CEL: carboxyethyl-lysine
CI: confidence intervals
CLF: collagen-linked fluorescence
CML: carboxymethyl-lysine
cMRI: cardiac magnetic resonance imaging
CT: computed tomography
CV: coefficient of variability
CVD: cardiovascular disease
DCCT: Diabetes Control and Complications Trial
EDIC: Epidemiology of Diabetes Interventions and Complications
ex/em: excitation/emission
G-H1: glyoxal hydroimidazolone
HbA_1c_: hemoglobin A1c
HPLC: high pressure liquid chromatography
HQC: high quality control
IMT: intima-media thickness
LC-MS/MS: liquid chromatography-tandem mass spectrometry
LQC: low quality control
LVM: left ventricular mass
LW-1: long wavelength fluorophore-1
LWF: long wavelength fluorescence
MA: microaneurysms
NCV/ANS: nerve conduction velocity/autonomic nervous system
MG-H1: methylglyoxal hydroimidazolone
NMR: nuclear magnetic resonance
NS: nonsignificant
QC: quality control
SAF: skin autofluorescence
SD: standard deviation
SIF: skin intrinsic fluorescence

## References

[1] Sell D, Monnier V (2012). Molecular basis of arterial stiffening: role of glycation—a mini-review. Gerontology.

[2] Monnier VM, Kohn RR, Cerami A (1984). Accelerated age-related browning of human collagen in diabetes mellitus. Proc Natl Acad Sci USA.

[3] Monnier VM, Vishwanath V, Frank KE, Elemts CA, Dauchot P, Kohn RR (1986). Relation between complications of type 1 diabetes mellitus and collagen-linked fluorescence. N Engl J Med.

[4] Thomas MC, Forbes JM, MacIsaac R, Jerums G, Cooper ME (2005). Low-molecular weight advanced glycation end products: markers of tissue AGE accumulation and more?. Ann NY Acad Sci.

[5] Kikugawa K, Beppu M (1987). Involvement of lipid oxidation products in the formation of fluorescent and crosslinked proteins. Chem Phys Lipids.

[6] Breunig HG, Studier H, König K (2010). Multiphoton excitation characteristics of cellular fluorophores of human skin in vivo. Opt Express.

[7] Croce AC, Ferrigno A, Piccolini VM, Tarantola E, Boncompagni E, Bertone V, Milanesi G, Freitas I, Vairetti M, Bottiroli G (2014). Integrated autofluorescence characterization of a modified-diet liver model with accumulation of lipids and oxidative stress. Biomed Res Int.

[8] Conway BN, Aroda VR, Maynard JD, Matter N, Fernandez S, Ratner RE, Orchard TJ (2011). Skin intrinsic fluorescence correlates with autonomic and distal symmetrical polyneuropathy in individuals with type 1 diabetes. Diabetes Care.

[9] Sugisawa E, Miura J, Iwamoto Y, Uchigata Y (2013). Skin autofluorescence reflects integration of past long-term glycemic control in patients with type 1 diabetes. Diabetes Care.

[10] Maynard JD, Rohrscheib M, Way JF, Nguyen CM, Ediger MN (2007). Noninvasive type 2 diabetes screening: superior sensitivity to fasting plasma glucose and A1C. Diabetes Care.

[11] Januszewski AS, Sachithanandan N, Karschimkus CS, O’Neal DN, Yeung CK, Alkatib N, Jenkins AJ (2012). Non-invasive measures of tissue autofluorescence are increased in type 1 diabetes complications and correlate with a non-invasive measure of vascular dysfunction. Diabet Med.

[12] den Engelsen C, van den Donk M, Gorter KJ, Salomé PL, Rutten GE (2012). Advanced glycation end products measured by skin autofluorescence in a population with central obesity. Dermatoendocrinol.

[13] Ueno H, Koyama H, Tanaka S, Fukumoto S, Shinohara K, Shoji T, Emoto M, Tahara H, Kakiya R, Tabata T (2008). Skin autofluorescence, a marker for advanced glycation end product accumulation, is associated with arterial stiffness in patients with end-stage renal disease. Metabolism.

[14] Fu M, Knecht K, Thorpe S, Baynes J (1992). Role of oxygen in cross-linking and chemical modification of collagen by glucose. Diabetes.

[15] Sell DR, Nemet I, Monnier VM (2010). Partial characterization of the molecular nature of collagen-linked fluorescence: role of diabetes and end-stage renal disease. Arch Biochem Biophys.

[16] Krolewski AS, Kosinski EJ, Warram JH, Leland OS, Busick EJ, Asmal AC, Rand LI, Christlieb AR, Bradley RF, Kahn CR (1987). Magnitude and determinants of coronary artery disease in juvenile-onset, insulin-dependent diabetes mellitus. Am J Cardiol.

[17] Cleary PA, Orchard TJ, Genuth S, Wong ND, Detrano R, Backlund JY, Zinman B, Jacobson A, Sun W, Lachin JM (2006). The effect of intensive glycemic treatment on coronary artery calcification in type 1 diabetic participants of the Diabetes Control and Complications Trial/Epidemiology of Diabetes Interventions and Complications (DCCT/EDIC) Study. Diabetes.

[18] Cleary PA, Braffett B, Orchard T, Lyons TJ, Maynard J, Cowie C, Gubitosi-Klug RA, Way J, Anderson K, Barnie A (2013). Clinical and technical factors associated with skin intrinsic fluorescence in subjects with type 1 diabetes from the Diabetes Control and Complications Trial/Epidemiology of Diabetes Interventions and Complications Study. Diabetes Technol Ther.

[19] Monnier VM, Bautista O, Kenny D, Sell DR, Fogarty J, Dahms W, Cleary PA, Lachin J, Genuth S (1999). Skin collagen glycation, glycoxidation and crosslinking are lower in subjects with long-term intensive versus conventional therapy of type 1 diabetes: relevance of glycated collagen products versus HbA1c as markers of diabetic complications. DCCT Skin Collagen Ancillary Study Group. Diabetes Control and Complications Trial. Diabetes.

[20] Monnier VM, Sell DR, Strauch C, Sun W, Lachin JM, Cleary PA, Genuth S (2013). DCCT Research Group The association between skin collagen glucosepane and past progression of microvascular and neuropathic complications in type 1 diabetes. J Diabetes Complications.

[21] The DCCT Research Group (1993). The effects of intensive treatment of diabetes on the development and progression of long-term complications in insulin-dependent diabetes mellitus. N Engl J Med.

[22] The DCCT/EDIC Research Group (2015). Effect of intensive diabetes therapy on the progression of diabetic retinopathy in patients with type 1 diabetes: 18 years of follow-up in the DCCT/EDIC. Diabetes.

[23] Fan X, Sell DR, Zhang J, Nemet I, Theves M, Lu J, Strauch C, Halushka MK, Monnier VM (2010). Anaerobic vs aerobic pathways of carbonyl and oxidant stress in human lens and skin during aging and in diabetes: a comparative analysis. Free Radic Biol Med.

[24] Stegeman H, Stalder S (1967). Determination of hydroxyproline. Clin Chim Acta.

[25] Thornalley PJ, Battah S, Ahmed N, Karachalias N, Agalou S, Babaei-Jadidi R, Dawnay A (2003). Quantitative screening of advanced glycation endproducts in cellular and extracellular proteins by tandem mass spectrometry. Biochem J.

[26] Orchard TJ, Lyons TJ, Cleary PA, Braffett BH, Maynard J, Cowie C, Gubitosi-Klug RA, Way J, Anderson K, Barnie A (2013). The association of skin intrinsic fluorescence with type 1 diabetes complications in the DCCT/EDIC study. Diabetes Care.

[27] Conway B, Edmundowicz D, Matter N, Maynard J, Orchard T (2010). Skin fluorescence correlates strongly with coronary artery calcification severity in type 1 diabetes. Diabetes Technol Ther.

[28] Hull EL, Ediger MN, Unione AHT, Deemer EK, Stroman ML, Baynes JW (2004). Noninvasive, optical detection of diabetes: model studies with porcine skin. Opt Express.

[29] Noordzij MJ, Lefrandt JD, Graaff R, Smit AJ (2011). Dermal factors influencing measurement of skin autofluorescence. Diabetes Technol Ther.

[30] The DCCT/EDIC Research Group (2003). Intensive diabetes therapy and carotid intima-media thickness in type 1 diabetes mellitus. N Engl J Med.

[31] Turkbey EB, Backlund JY, Genuth S, Jain A, Miao C, Cleary PA, Lachin JM, Nathan DM, van der Geest RJ, Soliman EZ (2011). Myocardial structure, function, and scar in patients with type 1 diabetes mellitus. Circulation.

[32] Genuth S, Sun W, Cleary P, Gao X, Sell DR, Lachin J (2015). The DCCT/EDIC Research Group, Monnier VM. Skin advanced glycation endproducts (AGEs) glucosepane and methylglyoxal hydroimidazolone are independently associated with long-term microvascular complication progression of type I diabetes. Diabetes.

[33] Hofmann B, Adam AC, Jacobs K, Riemer M, Erbs C, Bushnaq H, Simm A, Silber RE, Santos AN (2013). Advanced glycation end product associated skin autofluorescence: a mirror of vascular function?. Exp Gerontol.

[34] Pageon H, Zucchi H, Rousset F, Monnier VM, Asselineau D (2014). Skin aging by glycation: lessons from the reconstructed skin model. Clin Chem Lab Med.

[35] Monnier VM, Sun W, Gao X, Sell DR, Cleary PA, Lachin JM, Genuth S (2015). The DCCT/EDIC Research Group. Skin collagen advanced glycation endproducts (AGEs) and the long-term progression of sub-clinical cardiovascular disease in type 1 diabetes. Cardiovasc Diabetol.

[36] Balla S, Nusair MB, Alpert MA (2013). Risk factors for atherosclerosis in patients with chronic kidney disease: recognition and management. Curr Opin Pharmacol.

[37] Zoccali C, Benedetto FA, Mallamaci F, Tripepi G, Giacone G, Cataliotti A, Seminara G, Stancanelli B, Malatino LS (2001). CREED Investigators. Prognostic impact of the indexation of left ventricular mass in patients undergoing dialysis. J Am Soc Nephrol.

[38] Conway BN, Aroda VR, Maynard JD, Matter N, Fernandez S, Ratner RE, Orchard TJ (2012). Skin intrinsic fluorescence is associated with coronary artery disease in individuals with long duration of type 1 diabetes. Diabetes Care.

[39] Meerwaldt R, Links TP, Graaff R, Hoogenberg K, Lefrandt JD, Baynes JW, Gans RO, Smit AJ (2005). Increased accumulation of skin advanced glycation end-products precedes and correlates with clinical manifestation of diabetic neuropathy. Diabetologia.

[40] Hu H, Han CM, Hu XL, Ye WL, Huang WJ, Smit AJ (2012). Elevated skin autofluorescence is strongly associated with foot ulcers in patients with diabetes: a cross-sectional, observational study of Chinese subjects. J Zhejiang Univ Sci B.

[41] Chabroux S, Canouï-Poitrine F, Reffet S, Mills-Joncour G, Morelon E, Colin C, Thivolet C (2010). Advanced glycation end products assessed by skin autofluorescence in type 1 diabetics are associated with nephropathy, but not retinopathy. Diabetes Metab.

[42] Gerrits EG, Lutgers HL, Kleefstra N, Graaff R, Groenier KH, Smit AJ, Gans RO, Bilo HJ (2008). Skin autofluorescence: a tool to identify type 2 diabetic patients at risk for developing microvascular complications. Diabetes Care.

[43] Genuth S, Sun W, Cleary P, Sell DR, Dahms W, Malone J, Sivitz W, Monnier VM (2005). DCCT Skin Collagen Ancillary Study Group. Glycation and carboxymethyllysine levels in skin collagen predict the risk of future 10-year progression of diabetic retinopathy and nephropathy in the diabetes control and complications trial and epidemiology of diabetes interventions and complications participants with type 1 diabetes. Diabetes.

[44] Meerwaldt R, Hartog JW, Graaff R, Huisman RJ, Links TP, den Hollander NC, Thorpe SR, Baynes JW, Navis G, Gans RO (2005). Skin autofluorescence, a measure of cumulative metabolic stress and advanced glycation end products, predicts mortality in hemodialysis patients. J Am Soc Nephrol.

[45] Aroda VR, Conway BN, Fernandez SJ, Matter NI, Maynard JD, Orchard TJ, Ratner RE (2013). Cross-sectional evaluation of noninvasively detected skin intrinsic fluorescence and mean hemoglobin A1C in type 1 diabetes. Diabetes Technol Ther.

[46] Noordzij MJ, Mulder DJ, Oomen PHN, Brouwer T, Jager J, Castro Cabezas M, Lefrandt JD, Smit AJ (2012). Skin autofluorescence and risk of micro- and macrovascular complications in patients with type 2 diabetes mellitus—a multi-centre study. Diabet Med.

[47] Bos DC, de Ranitz-Greven WL, de Valk HW (2011). Advanced glycation end products, measured as skin autofluorescence and diabetes complications: a systematic review. Diabetes Technol Ther.

[48] Noordzij MJ, Lefrandt JD, Loeffen EAH, Saleem BR, Meerwaldt R, Lutgers HL, Smit AJ, Zeebregts CJ (2012). Skin autofluorescence is increased in patients with carotid artery stenosis and peripheral artery disease. Int J Cardiovasc Imaging.

[49] Meerwaldt R, Graaff R, Oomen PH, Links TP, Jager JJ, Alderson NL, Thorpe SR, Baynes JW, Gans RO, Smit AJ (2004). Simple non-invasive assessment of advanced glycation endproduct accumulation. Diabetologia.

[50] Eny KM, Lutgers HL, Maynard J, Klein BE, Lee KE, Atzmon G, Monnier VM, van Vliet-Ostaptchouk JV, Graaff R, van der Harst P (2014). GWAS identifies an NAT2 acetylator status tag single nucleotide polymorphism to be a major locus for skin fluorescence. Diabetologia.

[51] Roshandel D, Klein R, Klein B, Wolffenbuttel H, Atzmon G, Crandall J, Barzilai N, Bull S, Canty A, Hosseini S et al. A locus associated with skin intrinsic fluorescence in type 1 diabetes. In press. 2016. 10.2337/db15-1484 PMC4915582 27207532

[52] Eny KM, Orchard TJ, Miller RG, Maynard J, Grant DM, Costacou T, Cleary PA, Braffett BH, Paterson AD, The DCCT/EDIC Research Group (2015). Caffeine consumption contributes to skin intrinsic fluorescence in type 1 diabetes. Diabetes Technol Ther.

