# Supplementary material for: Skin collagen fluorophore LW-1 versus skin fluorescence as markers for the long-term progression of subclinical macrovascular disease in type 1 diabetes
Source: Cardiovasc Diabetol. 2016 Feb 11;15:30. doi: 10.1186/s12933-016-0343-3 (PMC4750185; doi:10.1186/s12933-016-0343-3)

ADDITIONAL FILE 1

Additional file 1 HPLC-fluorescence chromatograms of LW-1 eluting from a 15 cm X 2.1 mm, 3 µm Discovery HS C18 column. For the standard, LW-1 ~ 6 pmoles was injected in a 10 µl volume. For DCCT samples, including the nondiabetic, digests of insoluble collagen prepared from skin biopsies were injected onto the HPLC in 14 µl volumes, each containing ~ 61 to 69 µg collagen. The low and high quality controls (LQC and HQC) consists of collagen digests prepared from autopsied skin samples from a 22 and 38 year-old patient, respectively (see Methods). The former died from meningitis while the latter died from ESRD secondary to type 1 diabetes. Totals of 64 µg collagen in 10 µl were injected onto the HPLC column. Peaks eluting > 100 minutes represent column washing and equilibration (see Methods).

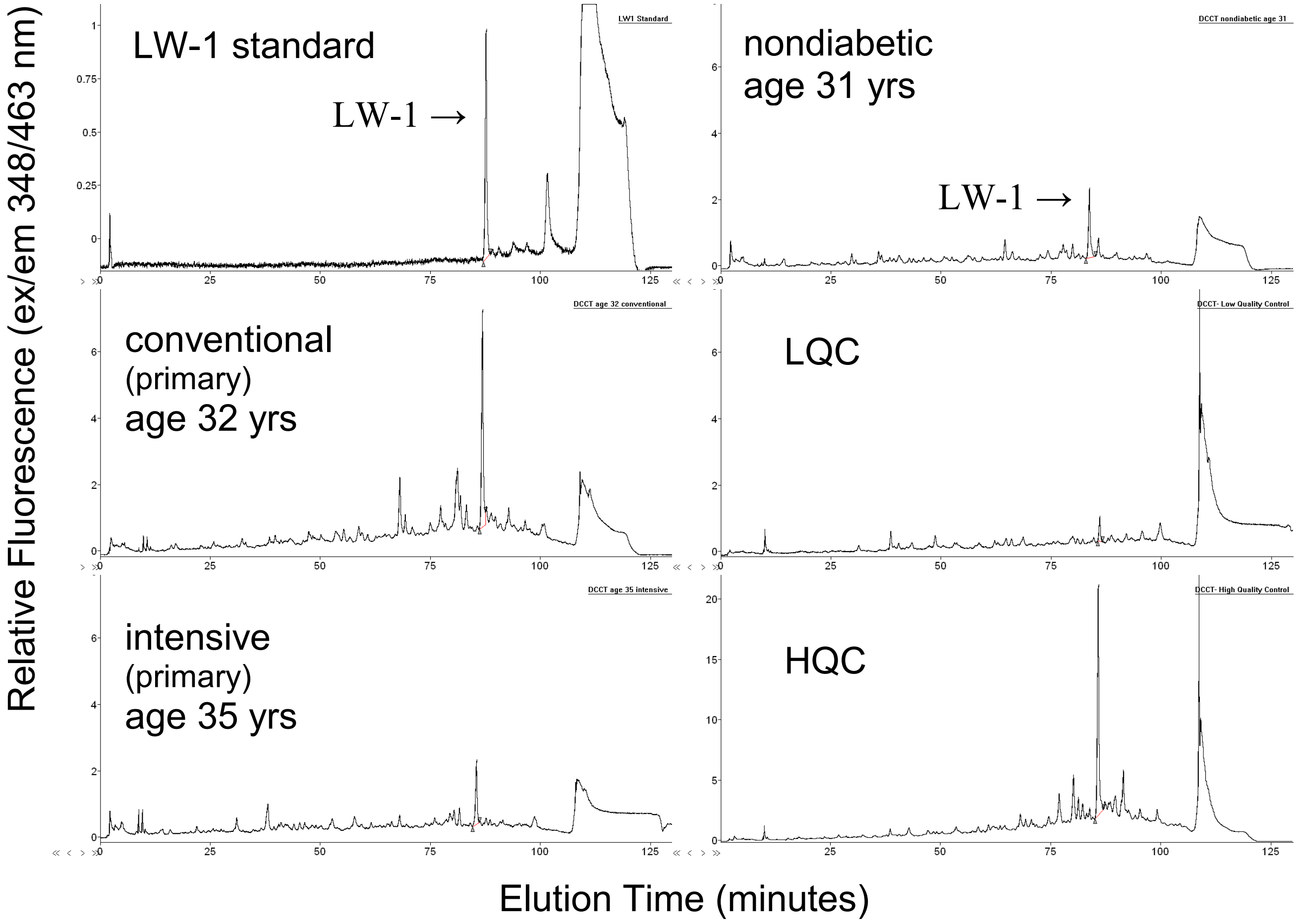

Supplement: Supplementary file 1 — 10.1186/s12933-016-0343-3 HPLC-fluorescence chromatograms of LW-1 eluting from a 15 cm X 2.1 mm, 3 μm Discovery HS C18 column. For the standard, LW-1 ~ 6 pmoles was injected in a 10 μl volume. For DCCT samples, including the nondiabetic, digests of insoluble collagen prepared from skin biopsies were injected onto the HPLC in 14 μl volumes, each containing ~ 61 to 69 μg collagen. The low and high quality controls (LQC and HQC) consists of collagen digests prepared from autopsied skin samples from a 22 and 38 year-old patient, respectively (see Methods). The former died from meningitis while the latter died from ESRD secondary to type 1 diabetes. Totals of 64 μg collagen in 10 μl were injected onto the HPLC column. Peaks eluting > 100 minutes represent column washing and equilibration (see "Methods" section). [file 12933_2016_343_MOESM1_ESM.pdf]
