# Supplementary material for: Skin collagen fluorophore LW-1 versus skin fluorescence as markers for the long-term progression of subclinical macrovascular disease in type 1 diabetes
Source: Cardiovasc Diabetol. 2016 Feb 11;15:30. doi: 10.1186/s12933-016-0343-3 (PMC4750185; doi:10.1186/s12933-016-0343-3)

ADDITIONAL FILE 2

Additional file 2 Levey-Jennings plot for LW-1 assay in the DCCT study. The plot consists of injection number on the x-axis (i.e., from 1 to 357 in consecutive order over the six week course of the HPLC assay) *vs*. integrated peak areas on the y-axis (relative fluorescence at ex/em 348/463 nm) for all samples injected (n=216 DCCT + n=42 nondiabetic controls + n=13 repeats), LQC (n=13), HQC (n=14), LW-1 QC standard (n=49) and enzyme blanks (n=10).

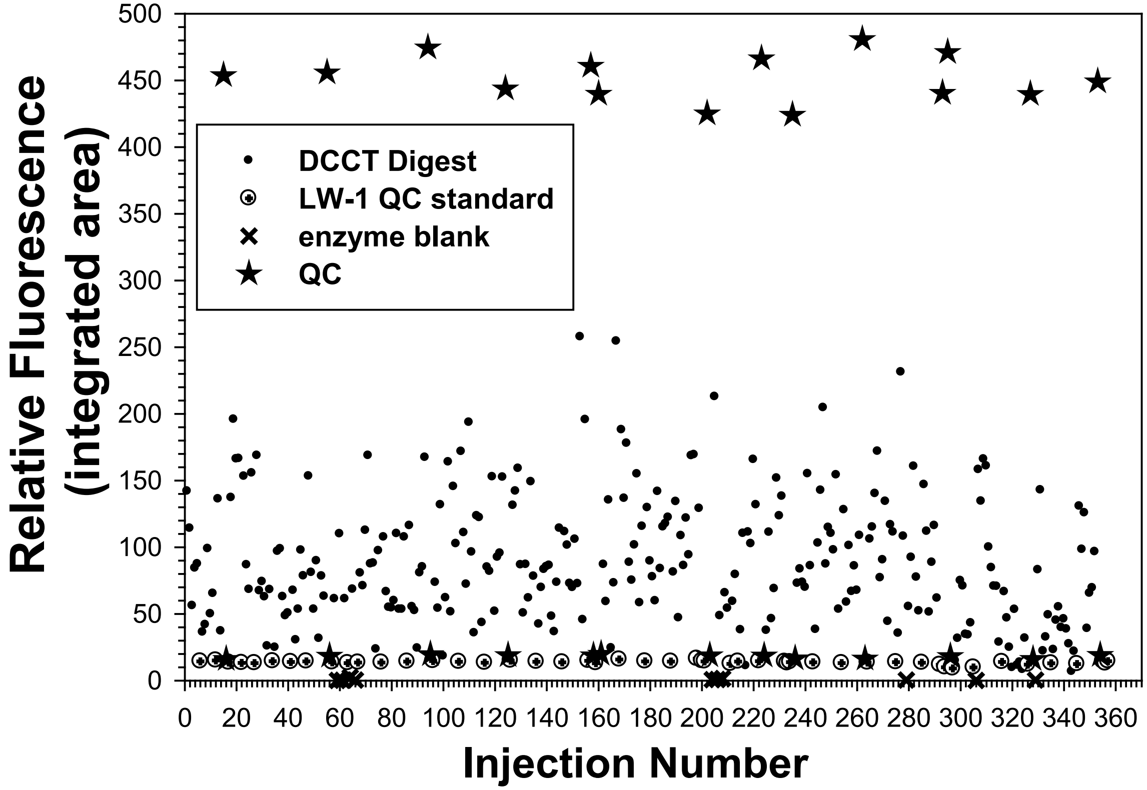

Supplement: Supplementary file 2 — 10.1186/s12933-016-0343-3 Levey-Jennings plot for LW-1 assay in the DCCT study. The plot consists of injection number on the x-axis (i.e., from 1 to 357 in consecutive order over the six week course of the HPLC assay) vs. integrated peak areas on the y-axis (relative fluorescence at ex/em 348/463 nm) for all samples injected (n = 216 DCCT + n = 42 nondiabetic controls + n = 13 repeats), LQC (n = 13), HQC (n = 14), LW-1 QC standard (n = 49) and enzyme blanks (n = 10). [file 12933_2016_343_MOESM2_ESM.pdf]
