# Supplementary material for: Skin collagen fluorophore LW-1 versus skin fluorescence as markers for the long-term progression of subclinical macrovascular disease in type 1 diabetes
Source: Cardiovasc Diabetol. 2016 Feb 11;15:30. doi: 10.1186/s12933-016-0343-3 (PMC4750185; doi:10.1186/s12933-016-0343-3)

ADDITIONAL FILE 3

Additional file 3 LW-1 levels increases with age in insoluble skin collagen of DCCT participants at DCCT closeout (x:age, y:LW-1). *controls:* subjects without diabetes, regression line & 95% confidence intervals (CI) of prediction, y=0.05 + 7x, r=0.64, P<0.0001, n=42. *conventional:* regression line (dash line) y=21+ 13x, r=0.43, P<0.0001, n=94. *intensive:* regression line (dash line) y=11x – 22, r=0.42, P<0.0001, n=122. Regression line & 95% CI for the controls have been reproduced in the latter two graphs for comparison. Symbols used: treatment (cohort) □, controls; ○, conventional (primary); Δ, conventional (secondary); ●, intensive (primary); ▲ intensive (secondary).

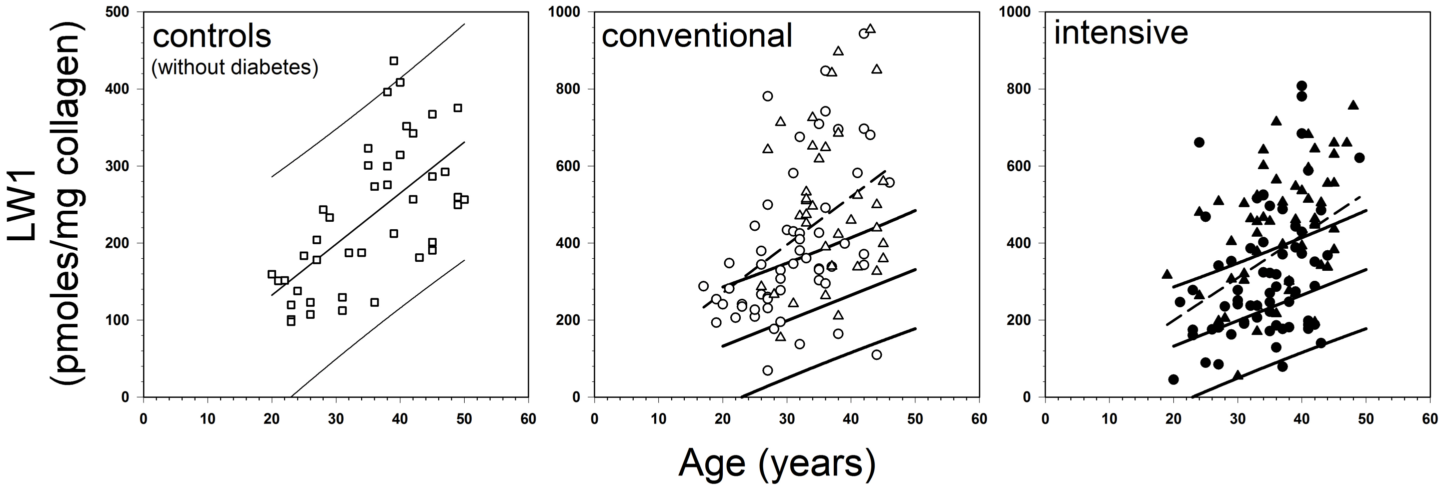

Supplement: Supplementary file 3 — 10.1186/s12933-016-0343-3 LW-1 levels increase with age in insoluble skin collagen of DCCT participants at DCCT closeout (x: age, y: LW-1). Controls: subjects without diabetes, regression line and 95 % CI prediction, y = 0.05 + 7x, r = 0.64, P < 0.0001, n = 42. Conventional: regression line (dash line) y = 21 + 13x, r = 0.43, P < 0.0001, n = 94. Intensive: regression line (dash line) y = 11x - 22, r = 0.42, P < 0.0001, n = 122. The regression line and 95 % CI for the controls have been reproduced in the latter two graphs for comparison. Symbols used: treatment (cohort): squares, controls; circles, conventional (primary); triangle, conventional (secondary); filled circle, intensive (primary); filled triangle, intensive (secondary) [file 12933_2016_343_MOESM3_ESM.pdf]
