# Supplementary material for: Skin collagen fluorophore LW-1 versus skin fluorescence as markers for the long-term progression of subclinical macrovascular disease in type 1 diabetes
Source: Cardiovasc Diabetol. 2016 Feb 11;15:30. doi: 10.1186/s12933-016-0343-3 (PMC4750185; doi:10.1186/s12933-016-0343-3)

ADDITIONAL FILE 4

Additional File 4 Plots of unadjusted LW-1 levels *vs.* AGEs measured in insoluble skin collagen in the combined dataset; i.e., DCCT (n=216) plus nondiabetic controls (n=42). Levels of all measured parameters except collagen insolubility (%) and CLF (arbitrary fluorescence units/mg collagen) are stated in picomoles per mg collagen. For each graph, the linear regression line and 95% confidence intervals of prediction determined for the combined dataset are shown along with its correlation coefficient and statistical significance (P-value). Symbols used: treatment (cohort) □, controls; ○, conventional (primary); Δ, conventional (secondary); ●, intensive (primary); ▲ intensive (secondary).

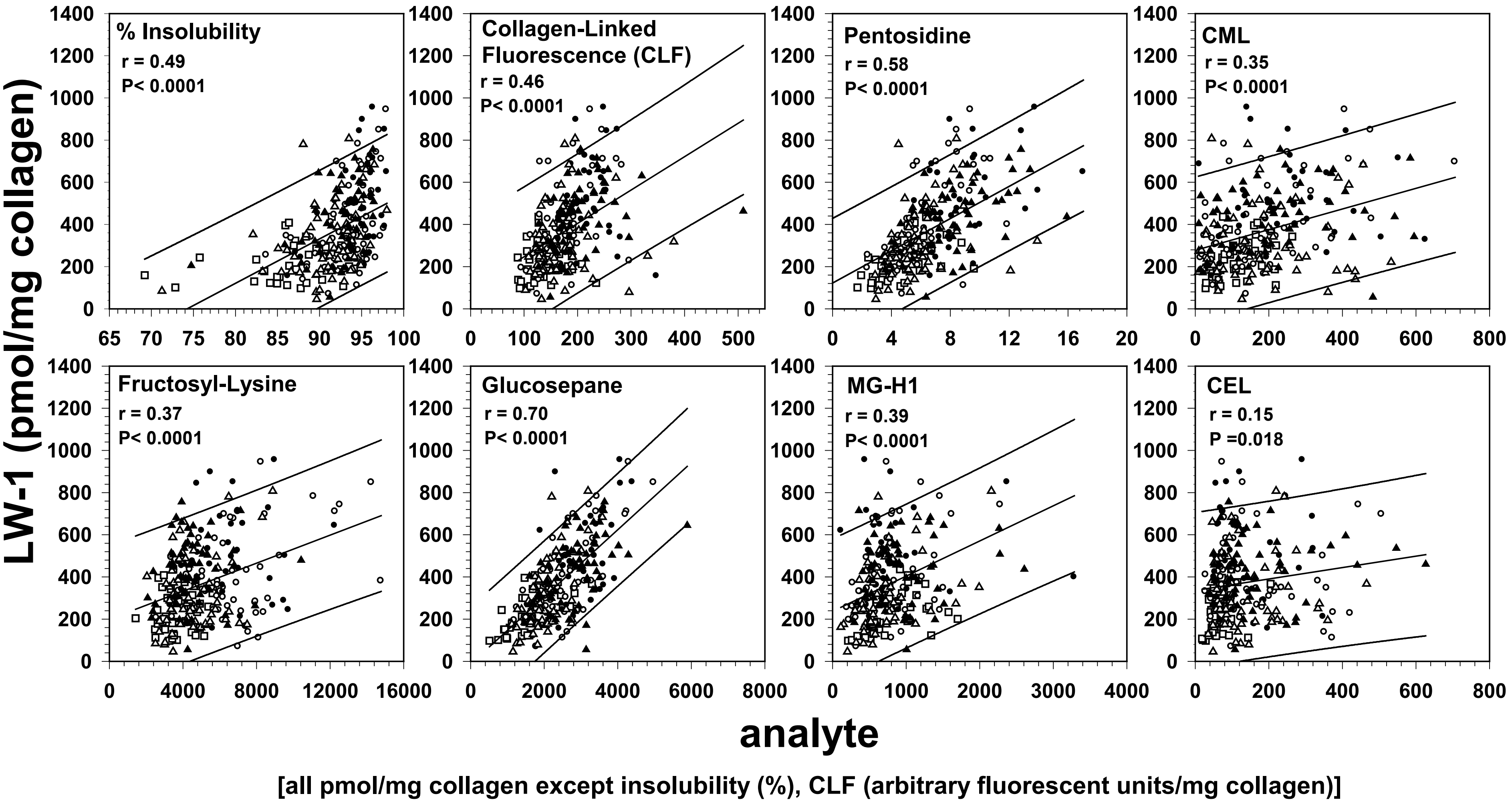

Supplement: Supplementary file 4 — 10.1186/s12933-016-0343-3 Plots of unadjusted LW-1 levels vs. AGEs measured in insoluble skin collagen in the combined dataset: i.e., DCCT (n = 216) plus nondiabetic controls (n = 42). Levels of all measured parameters except collagen insolubility (%) and CLF (arbitrary fluorescence units/mg collagen) are stated in picomoles per mg collagen. For each graph, the linear regression line and 95 % CI prediction determined for the combined dataset are shown along with its correlation coefficient and statistical significance (P value). Symbols used: see Additonal file 3. [file 12933_2016_343_MOESM4_ESM.pdf]
