# Supplementary material for: Skin collagen fluorophore LW-1 versus skin fluorescence as markers for the long-term progression of subclinical macrovascular disease in type 1 diabetes
Source: Cardiovasc Diabetol. 2016 Feb 11;15:30. doi: 10.1186/s12933-016-0343-3 (PMC4750185; doi:10.1186/s12933-016-0343-3)
Supplement: Supplementary file 5 — 10.1186/s12933-016-0343-3 Partial correlation analysis of LW-1 with other skin collagen markers controlling for age and duration of diabetes. [file 12933_2016_343_MOESM5_ESM.pdf]

ADDITIONAL FILE 5

Additional file 5 Partial correlation analysis of LW-1 with other skin collagen markers controlling for age and duration of diabetes.* †

LW-1	Age (years)‡	Duration (months)§	Glucosepane	Pentosidine	Fructosyl -lysine	MG-H1	CML	G-H1	CEL	Pepsin Solubility	Acid Solubility	Collagen-Linked Fluorescence (CLF)‖	Skin Intrinsic Fluorescence (SIF) SCOUT DS‖
Correlation (r)	0.35	0.15	0.60	0.44	0.40	0.34	0.24	0.16	0.04	-0.38	-0.13	0.27	0.13
Significance (P value)	<0.0001	0.026	<0.0001	<0.0001	<0.0001	<0.0001	<0.0001	0.024	NS	<0.0001	0.052 NS	<0.0001	0.084 NS

* Correlations made over combined DCCT cohorts, n=216; SIF, n=185.
† NS: nonsignificant (P>0.05).
‡ controlling for duration
§ controlling for age
‖ CLF was measured in the same skin biopsy (buttock) as LW-1 (n=216) at DCCT closeout (1992-1993) while SIF was noninvasively measured by the SCOUT DS on the volar forearm skin in the same DCCT/EDIC cohort (n=185) at EDIC years 16-17 (2009-2010). See Methods.
