# Supplementary material for: Skin collagen fluorophore LW-1 versus skin fluorescence as markers for the long-term progression of subclinical macrovascular disease in type 1 diabetes
Source: Cardiovasc Diabetol. 2016 Feb 11;15:30. doi: 10.1186/s12933-016-0343-3 (PMC4750185; doi:10.1186/s12933-016-0343-3)
Supplement: Supplementary file 6 — 10.1186/s12933-016-0343-3 LW-1 correlates better with recent rather than long-term cumulative glycemia. Univariate regression models of age- and duration-adjusted LW-1 (biopsy) vs. HbA1c at different sampling times in the DCCT. [file 12933_2016_343_MOESM6_ESM.pdf]

ADDITIONAL FILE 6

Additional file 6 LW-1 correlates better with recent rather than long-term cumulative glycemia. Univariate regression models of age- and duration-adjusted LW-1 (biopsy) *vs.* HbA_{1c} at different sampling times in the DCCT.

HbA_{1c} Sampling Time	Model R^2 (%)	P-Value
Initial screening	2.7	0.0149
Mean up to biopsy (~7-10 years)	9.9	<0.0001
Mean over the past 1 year	9.6	<0.0001
Nearest to biopsy	10.1	<0.0001
